# Testicular sperm aspiration has a poor effect in predicting micro-TESE outcomes in NOA patients with AZFc deletion

**DOI:** 10.1186/s12610-023-00195-x

**Published:** 2023-08-10

**Authors:** Chenyao Deng, Jiaming Mao, Lianming Zhao, Defeng Liu, Haocheng Lin, Zhe Zhang, Yuzhuo Yang, Haitao Zhang, Kai Hong, Hui Jiang

**Affiliations:** 1 Department of Urology, Peking University Third Hospital, Beijing, 100191 China; 2 Department of Andrology, Peking University Third Hospital, Beijing, 100191 China; 3 Department of Reproductive Medicine Center, Peking University Third Hospital, Beijing, 100191 China; 4 Department of Human Sperm Bank, Peking University Third Hospital, Haidian District, 49 North Garden Road, Beijing, 100191 China

**Keywords:** AZFc deletion, Nonobstructive azoospermia, Micro-TESE, Testicular sperm aspiration, Sperm retrieval rate, Délétion AZFc, Azoospermie non obstructive, Micro-TESE, Aspiration de spermatozoïdes testiculaires, Taux de récupération de spermatozoïdes, Délétion AZFc, Azoospermie non obstructive, Micro-TESE, Aspiration de spermatozoïdes testiculaires, Taux de récupération de spermatozoïdes

## Abstract

**Background:**

Testicular sperm aspiration (TESA) is widely used in the diagnosis and management of nonobstructive azoospermia. However, its ability for predicting microdissection testicular sperm extraction in nonobstructive azoospermia (NOA) patients with AZFc deletion remains uncertain. To investigate whether TESA affected the sperm retrieval rate (SRR) in NOA patients with AZFc deletion, a retrospective analysis of the clinical data of NOA patients with AZFc deletion who underwent microdissection testicular sperm extraction (micro-TESE) was conducted. The effects of age, testicular volume, follicle-stimulating hormone (FSH) levels, luteinizing hormone (LH) levels, testosterone (T) levels and TESA on the SRR were analyzed in this group of patients.

**Results:**

A total of 181 individuals had their sperm successfully collected and underwent micro-TESE, with an SRR of 67.4%. The patients were separated into two groups based on their micro-TESE results (sperm acquisition and nonsperm acquisition), with no significant variations in age, testicular volume, FSH levels, LH levels, or T levels between the two groups. There was no significant difference in the SRR between any of the groups into which patients were classified based on reproductive hormone reference value ranges. Binary logistic regression was used to explore the absence of significant effects of age, testicular volume, FSH levels, LH levels, and T levels on sperm acquisition in patients undergoing micro-TESE. In the preoperative testicular diagnostic biopsy group, the sperm acquisition and nonsperm acquisition groups had SRRs of 90.1% and 65.1%, respectively. More significantly, there was no significant difference in the SRR between the negative preoperative testicular diagnostic biopsy group and the nonpreoperative testicular diagnostic biopsy group (65.1 vs. 63.8%, *p* = 0.855).

**Conclusion:**

There is a high probability of successful sperm acquisition in the testis of men undergoing micro-TESE. In this group of patients, age, testicular volume, FSH levels, LH levels, and T levels may have little bearing on the micro-TESE outcome. In patients whose preoperative TESA revealed the absence of sperm, the probability of obtaining sperm by micro-TESE remained high (65.1%); negative TESA results appeared to not influence the SRR (63.8%) in patients undergoing micro-TESE.

## Résumé

**Contexte:**

L’aspiration testiculaire de spermatozoïdes (TESA) est largement utilisée dans le diagnostic et la prise en charge de l’azoospermie non obstructive. Cependant, sa capacité à prédire la présence de spermatozoïdes testiculaires lors de l’extraction par microdissection chez les patients atteints d’azoospermie non obstructive (NOA) et porteurs de la délétion AZFc reste incertaine. Pour déterminer si la TESA affectait le taux de récupération de spermatozoïdes (SRR) chez les patients atteints d’ANO avec délétion AZFc, nous avons mené une analyse rétrospective des données cliniques des patients atteints de NOA et d’une délétion AZFc ayant subi une extraction testiculaire de spermatozoïdes (micro-TESE) par microdissection. Les effets de l’âge, du volume testiculaire, des taux d’hormone folliculostimulante (FSH), d’hormone lutéinisante (LH), de testostérone (T) et de TESA sur le SRR ont été analysés chez ces patients.

**Résultats:**

Au total, 181 personnes ont eu leur spermatozoïdes collectés avec succès par micro-TESE, avec un SRR de 67,4%. Les patients ont été répartis en 2 groupes en fonction de leurs résultats à la micro-TESE (obtention de spermatozoïdes et non obtention de spermatozoïdes), sans variations significatives de l’âge, du volume testiculaire, des taux de FSH, LH ou de T entre les 2 groupes. Aucune différence significative du SRR n’a été retrouvée entre les groupes dans lesquels les patients ont été classés en fonction des plages de valeurs de référence des hormones reproductives. La régression logistique binaire a été utilisée pour explorer l’absence d’effets significatifs de l’âge, du volume testiculaire, des taux de FSH, de LH et de T sur la récupération de spermatozoïdes chez les patients bénéficiant d’une micro-TESE. Dans le groupe de biopsie diagnostique testiculaire préopératoire, les groupes avec récupération de spermatozoïdes et sans récupération de spermatozoïdes avaient respectivement des SRR de 90,1% et 65,1%. Plus important encore, il n’y avait pas de différence significative du SRR entre le groupe de biopsie diagnostique testiculaire préopératoire négatif et le groupe sans biopsie diagnostique testiculaire préopératoire (65,1 vs 63,8%, *p* = 0,855).

**Conclusions:**

Il existe une forte probabilité de récupération réussie de spermatozoïdes testiculaires chez les hommes bénéficiant d’une micro-TESE. Dans ce groupe de patients, l’âge, le volume testiculaire, les taux de FSH, de LH et de T ont peu d’incidence sur le résultat de la micro-TESE. Chez les patients dont la TESA préopératoire a révélé l’absence de spermatozoïdes, la probabilité d’obtenir des spermatozoïdes par micro-TESE est restée élevée (65,1%); les résultats négatifs d’une TESA ne semblaient pas influencer le SRR (63,8%) chez les patients bénéficiant d’une micro-TESE.

## Introduction

Up to 30% of male infertility is due to spermatogenic dysfunction caused by genetic abnormalities, particularly severe male infertility such as azoospermia and severe oligospermia [1]. Klinefelter syndrome is the most frequent genetic cause of male infertility, followed by azoospermia factor (AZF) deletion on the Y chromosome. The incidence of AZF deletion is approximately 4% in the general population and 10–15% in individuals with azoospermia [2]. Azoospermia is the most devastating kind of infertility in men. These men can only have biological children through intracytoplasmic sperm injection (ICSI), which involves surgically extracting mature sperm from the testes. Infertile couples can, of course, abandon surgical therapy in favor of donor sperm.

Testicular sperm extraction can be performed using a variety of surgical techniques. Both TESA and micro-TESE are frequently employed in clinical treatment, and each has its own set of benefits and drawbacks. It is critical to evaluate not only the surgical success rate but also a patient’s financial situation, their risk of complications, and their medical history when making clinical treatment decisions. TESA involves percutaneous aspiration of testicular tissue to obtain sperm, which is minimally invasive but somewhat blind, as the area of high-quality spermatogenesis may be unevenly distributed, resulting in a low number or poor quality of retrieved sperm or even failure to retrieve sperm [3]. Micro-TESE has a greater SRR than TESA and conventional TESE; however, the latter requires a longer processing time and is more expensive [4].

The hypothalamic-pituitary–testicular axis controls spermatogenesis, which is a complicated process. As a result, any of the hormones involved in this axis, such as follicle-stimulating hormone (FSH), luteinizing hormone (LH) and testosterone (T), might affect proper spermatogenesis. Other relevant factors, such as body mass index, prolactin levels, and estradiol levels, were shown to not influence this axis in most studies and were thus excluded from our study [5].

Noninvasive markers, such as a patient’s age, testicular volume, and reproductive hormone levels, as well as invasive indicators, such as testicular biopsy and testicular pathology reports, are useful for NOA patients planning to undergo micro-TESE [6, 7]. These can assist clinicians and infertile couples in making the best clinical decision possible. However, the measurements’ predictive usefulness is unclear, and while micro-TESE has been promoted as the gold standard for sperm retrieval in males with NOA, its superiority to conventional TESE is controversial [8, 9].

We retrospectively analyzed data from our center to investigate the effects of age, testicular volume, reproductive hormone levels, and TESA on sperm retrieval after micro-TESE in patients with NOA due to AZFc deletion.

## Materials and methods

### Patients

From January 2015 to December 2019, clinical data on age, testicular volume, reproductive hormone levels, and testicular sperm aspiration, as well as clinical outcomes regarding sperm found intraoperatively, were collected for NOA patients with AZFc deletion who underwent micro-TESE at our Center for Reproductive Medicine. For the analysis, the average volume of both testicles was employed (testometer used to measure bilateral tests). If the patient only had one testicle, only one testicle volume was utilized in the calculation. This was a retrospective single-center study. Patients who met the following criteria were excluded: 1) previous varicocele; 2) cryptorchidism; 3) history of radiotherapy; 4) Klinefelter or Kallman syndromes; 5) hypothalamic/pituitary defects; and 6) the use of medications that affect hormone levels (e.g., exogenous testosterone, selective estrogen receptor modulators, gonadotropins, or aromatase inhibitors).

Patients were divided into groups based on preoperative indicators. Based on the mean testicular volume, they were divided into 3 groups: < 6 mL (small testicle) and ≥ 6 mL. According to FSH levels, patients were divided into 3 groups: < 11.1 mIU/mL (normal reference value), 11.1–22.2 mIU/mL (2 times the upper limit of the normal reference value), and ≥ 22.2 mIU/mL (more than 2 times the upper limit of the normal reference value). According to LH levels, patients were divided into 2 groups: ≤ 6.8 mIU/mL (normal reference value) and ≥ 6.8 mIU/mL (above the upper limit of the reference value). According to T levels, patients were divided into 3 groups: < 8 nmol/L (a low testosterone level), 8–12 nmol/L (a suspiciously low testosterone level), and ≥ 12 nmol/L (a normal testosterone level).

### Methods

According to the European Academy of Andrology (EAA) and the European Molecular Genetics Quality Network (EMQN) guidelines for the molecular diagnosis of Y-chromosomal microdeletions, a total of eight loci were examined, sY14 (SRY), ZFX/ZFY, sY84, sY86, sY127, sY134, sY254, and sY255, of which both sY254 and sY255 were lacking due to AZFc deletion [10]. All of the patients underwent testing for the AZF gene and were clinically diagnosed with AZFc deletion, as well as chromosomal karyotyping to rule out chromosomal abnormalities.

The clinical diagnosis was nonobstructive azoospermia and AZFc deletion based on the patient’s history, physical examination, and pertinent laboratory test results. The 5th edition of the World Health Organization guidelines for semen analysis was used [11]. When no sperm were found in the semen after 15 min of centrifugation at 600 g in at least two tests, the patient was diagnosed with azoospermia. Before the operation, at least three semen analyses were performed, and after centrifugation, no sperm were discovered on microscopic examination. Before the operation, patients masturbated once more to gather semen for evaluation. Before microscopic sperm extraction, no sperm were detected in the semen, and if sperm was found in the semen, the procedure was canceled. Patients and their families were given information regarding surgery alternatives and risks, as well as informed consent forms to complete. Each patient had venous blood samples taken after an overnight fast (7 am to 11 am). A chemiluminescent immunoassay was used to quantify T levels (reference range > 12 nmol/L), luteinizing hormone levels (reference range = 0.6–6.8 mIU/mL), and follicle-stimulating hormone levels (reference range = 0.9–11.1 mIU/mL).

### Procedures

Some patients willingly choose to undergo TESA before micro-TESE, and depending on the results, they decided between micro-TESE and obtaining sperm from a sperm bank as their next course of therapy. At our center, the TESA procedure is divided into two parts: microscopy and histological analysis with sperm examination. The patient is placed in a lying position, and the doctor disinfects the skin in the perineal area with iodine and then applies local anesthesia with lidocaine. The skin of the scrotum is taut because one testicle is fixated by hand. The surgeon next inserts a piercing needle into the testis and pushes it back and forth to extract the testicular tissue. A sample of testicular tissue is placed in a culture medium, shipped to the lab to be ground, and then inspected under a microscope to check for the presence of spermatozoa to learn more about testicular spermatogenesis. Testicular histology is performed only to look for morphologically normal sperm in the sections and does not cost the patient additional money for pathological diagnosis. Finding morphologically normal sperm by either method is defined as a positive biopsy. Although TESA may lead to the identification of sperm, the patient may decide to proceed with micro-TESE to ensure that enough sperm is recovered for the next ICSI due to the insufficient number and quality of sperm.

The micro-TESE surgical procedure is described in a previous article published by Schlegel et al. [12]. A longitudinal incision is made in the middle of the scrotum, the skin and sheath are incised, and the testes and epididymis are exposed and extruded. Under an operating microscope (S88, Carl Zeiss, Germany) with a maximum magnification of 18x, the albuginea is incised along the middle transverse surface of the testis with a sharp blade, and the testicular parenchyma is carefully dissected layer by layer with miniature forceps. The relatively full and thick spermatogenic tubules are chosen, placed in a Petri plate, and delivered immediately to the embryo laboratory staff. During the operation, two senior laboratory staff members use a 1 mL syringe needle to slice, tear and separate testicular tissue to release spermatogenic cells and sperm from the germinal tubules to make a cell suspension that is examined under a microscope for mature sperm. If enough sperm with good morphology are detected, the surgery is completed. If no sperm is found in one testicle, the adjacent testicle is then incised and inspected thoroughly. Interrupted 5–0 silk sutures are used to seal the incision in the white membrane. The surgical incision is closed, the testis is moved back into the sheath, and the scrotum is bandaged tightly. When no spermatozoa are discovered intraoperatively in a patient, the obtained germinal tubules are maintained in culture for 24 h before another sperm search is conducted. If sperm are still not discovered, the germinal tubules are then cultured. In the absence of sperm, the culture fluid is centrifuged, and the search for sperm is repeated.

### Statistics

For statistical analysis, we used IBM SPSS Statistics version 25.0 (IBM Corporation, Armonk, NY, USA). The data were subjected to descriptive statistical analysis (the median as well as the interquartile range). To determine if the nonnormally distributed data differed between groups, the Wilcoxon test was performed. The variations in the SRR between groups were compared using the chi-square test and Fisher’s exact test. A significant difference was indicated by *p* < 0.05.

## Result

**Fig. 1:**
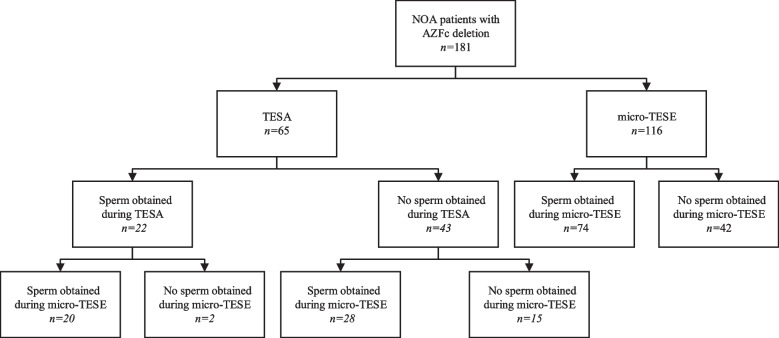
Flow chart of patients with AZFc deletion. AZF: azoospermia factor; TESA: testicular sperm aspiration; NOA: nonobstructive azoospermia; Micro-TESE: microdissection testicular sperm extraction

**Fig. 2:**
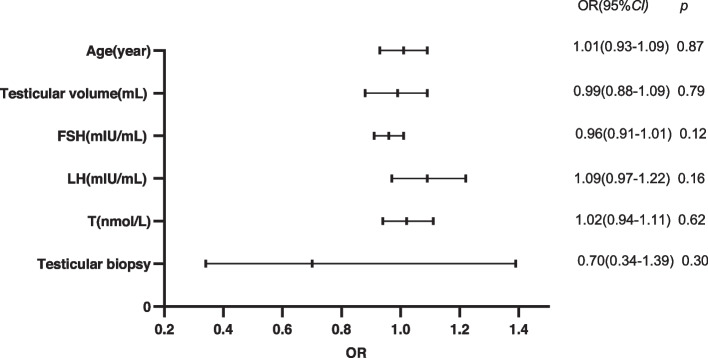
Results of binary logistic regression analysis. Age, testicular volume, reproductive hormone levels, and testicular biopsy results are not predictors of successful micro-TESE. FSH: follicle-stimulating hormone; LH: luteinizing hormone; T: testosterone; OR: odds ratio

A total of 181 NOA patients with AZFc deletion underwent micro-TESE, with sperm obtained for 122 men during treatment, resulting in an SRR of 67.4% (122/181). Sixty-five of these patients chose to undergo TESA prior to micro-TESE, and 22 had sperm found by TESA. Figure 1 illustrates the overall procedure. We compared baseline data on age, testicular volume, and serum reproductive hormone levels in patients in the micro-TESE sperm acquisition group (*n* = 122) and the nonsperm acquisition group (*n* = 59). There were no significant differences between the two groups in terms of age, testicular volume, FSH levels, LH levels, or T levels, as shown in Table 1. Age, testicular volume, reproductive hormone levels, whether TESA was performed were taken as the independent variables, and whether sperm were obtained during micro-TESE was taken as the dependent variable; this yielded ORs of 1.01, 0.99, 0.96, 1.09, 1.02, and 0.70, respectively, with no significant effects in the binary logistic regression analysis, as shown in Fig. 2. The SRR was separated into subgroups based on testicular volume and reproductive hormone levels, and the differences in the SRR between subgroups were compared. The SRRs of patients with testicular volumes > 6 mL and ≤ 6 mL in the two groups were compared, and there was no difference between the two groups. The SRRs of the groups with serum FSH levels < 11.1 mIU/mL, 11.2–22.2 mIU/mL and ≥ 22.2 mIU/mL were 75.0%, 67.0,% and 61.2%, respectively, with no difference among the groups; the SRRs of the groups with serum T levels < 8 nmol/L, 8–12 nmol/L and ≥ 12 nmol/L were 66.1%, 65.8,% and 71.4%, respectively, with no difference among the groups; the SRRs in the groups with serum LH levels < 6.8 mIU/mL and ≥ 6.8 mIU/mL were 64.4% and 70.3%, respectively, with no difference between the groups. Table 2 displays the results. The SRR of the final micro-TESE did not differ significantly across the groups, whether patients were divided by testicular volume or by reproductive hormone level.

**Table 1:** Patient baseline characteristics and comparison of age, testicular volume and reproductive hormone between two groups

Characteristics, median (IQR)	All patients (*n* = *181*)	Group1 (*n* = *122*)	Group2 (*n* = *59*)	*P*
Age (year)	30 (28–33)	30 (28–33)	30 (27–33)	0.797
Testicular volume (mL)	8 (6–12)	9 (7–12)	9 (7–11)	0.764
FSH (mIU/mL)	16.50 (11.10–16.50)	16.30 (10.70–22.13)	16.80 (11.80–24.20)	0.444
LH (mIU/mL)	6.80 (5.04–9.60)	6.95 (5.22–9.68)	6.47 (4.62–9.55)	0.365
T (nmol/L)	9.60 (7.68–12.45)	9.62 (7.70–12.55)	9.50 (7.38–11.60)	0.651

Non-parametric tests were used for comparisons between variables

*Group1* Sperm obtained during micro-TESE, *Group2* No sperm obtained during micro-TESE, *IQR* Interquartile range, *FSH* Follicle-stimulating hormone, *LH* Luteinizing hormone, *T* Testosterone

**Table 2:** Comparison of SRR between two groups

Subgroups	Group1 (*n* = *122*)	Group2 (*n* = *59*)	SRR	*P*
Testicular volume, *n*				
6 mL	30	17	63.8%	
≥ 6 mL	92	42	68.7%	0.54
FSH, *n*				
< 11.1 mIU/mL	33	11	75.0%	
11.1–22.2 mIU/mL	59	29	67.0%	
≥ 22.2 mIU/mL	30	19	61.2%	0.16
T, *n*				
< 8 nmol/L	37	19	66.1%	
8–12 nmol/L	50	26	65.8%	
≥ 12 nmol/L	35	14	71.4%	0.56
LH, *n*				
< 6.8 mIU/mL	58	32	64.4%	
≥ 6.8 mIU/mL	64	27	70.3%	0.40

Chi-square test is used for comparison between rates

*Group1* Sperm obtained during micro-TESE, *Group2* No sperm obtained during micro-TESE, *FSH* Follicle-stimulating hormone, *LH* Luteinizing hormone, *T* Testosterone, *SRR* Sperm retrieve rate

Sixty-five patients in our study underwent TESA, and some patients underwent intraoperative microscopy in conjunction with diagnostic testicular aspiration biopsy, during which a portion of the testicular tissue was torn apart and examined under a microscope to search for spermatozoa. Based on testicular biopsy results, we split the patients into two groups. For those who underwent postoperative testicular histology or intraoperative microscopy, 22 patients had mature spermatozoa visible, while 43 patients had no spermatozoa visible. Spermatozoa retrieval failed for only two of the 22 patients who underwent further micro-TESE, with an SRR of 90.1%. In contrast, 43 patients underwent later micro-TESE surgery, with 28 achieving a success rate of 65.1%, with a significant difference between the two groups (*p* = 0.036). Table 3 shows that there was no statistically significant difference in the SRR between the preoperative testicular diagnostic biopsy group and the nonpreoperative testicular diagnostic biopsy group (73.9 vs. 63.8%, *p* = 0.166). More interestingly, the SRR was very similar between the negative preoperative testicular diagnostic biopsy group and the nonpreoperative testicular diagnostic biopsy group, with no significant difference (65.1 vs. 63.8%, *p* = 0.855).

**Table 3:** Comparison of SRR between two groups

Factors	Group1 (*n* = *122*)	Group2 (*n* = *59*)	SRR	*P*
No testicular biopsy, *n*	72	42	63.8%	
Testicular biopsy, *n*	48	17	73.9%	0.166^a^
No obtain sperm, *n*	28	15	65.1%	0.855^b^
Obtain sperm, *n*	20	2	90.1%	0.036^c^

If the expected frequencies are all greater than 5, the pearson chi-square can be used directly at this point. If any of the expected frequencies are less than 5, the continuous calibration chi-square test can be used at this point

*Group1* Sperm obtained during micro-TESE, *Group2* No sperm obtained during micro-TESE, *SRR* Sperm retrieve rate

^a^Testicular biopsy vs. No testicular biopsy

^b^No obtain sperm vs. No obtain sperm

^c^No obtain sperm vs. Obtain sperm

## Discussion

AZFc deletion, the second most frequent genetic factor in NOA, is quite common in NOA patients [13]. Noninvasive indicators such as age, testicular size, and reproductive hormone levels, as well as invasive testicular biopsies, are important in determining the spermatogenic function of the testes before micro-TESE and can assist clinicians and infertile couples in making the best clinical decisions for them [14]. A study suggested that spermatogenic function declines with age in patients with AZFc deletion and that early assisted reproduction or sperm freezing is recommended for patients with AZFc deletion [15]. However, our study focused on NOA patients with AZFc deletion, and the results showed that age did not affect the SRR of micro-TESE. The findings of Caroppo et al. demonstrated that testicular volume and FSH levels did not affect the SRR in NOA patients, which is consistent with the findings of this study [16]. Our findings reveal that while the sperm acquisition rate decreased as FSH levels increased, there was no statistically significant difference between the groups. According to a systematic review of testicular pathology, in 19 studies with 178 patients with AZFc deletion, Sertoli-only cell syndrome accounted for 46% of the patients with a testicular pathology, inhibited maturation for 38%, and lead to hypospermatogenesis for 16% [17]. According to our clinical experience, spermatogenic function in the testes of patients with AZFc deletion is extremely heterogeneous, and the testes may contain three different pathological tissue types at the same time; therefore, a simple testicular pathological examination is rarely representative of the actual spermatogenic situation of the entire testis. The SRR for azoospermia patients with AZFc deletion who underwent fine needle aspiration (FNA) for sperm extraction was 34.7% (17/49), according to a study conducted at our center [18]. In this study, the SRR was 67.4% (122/181) in 181 NOA patients with AZFc deletion, nearly twice as high as that of traditional sperm retrieval via TESA. When comparing the obtained sperm and unobtained sperm groups, the preoperative testicular diagnostic biopsy group had an SRR of 90.1% and 65.1%, respectively. Although micro-TESE had a better chance of retrieving sperm in patients with sperm identified in a preoperative testicular biopsy, sperm retrieval failed for approximately 10% of patients. Micro-TESE, on the other hand, has a higher likelihood of retrieving mature sperm in patients whose preoperative testicular biopsy did not reveal sperm than in average patients; hence, the predictive utility of using preoperative testicular biopsy pathology results for subsequent micro-TESE is limited. For patients with NOA due to AZFc deletion, we advocate direct micro-TESE to maximize the likelihood of detecting sperm intraoperatively, obtain a sufficient number and quality of sperm for ICSI-assisted conception and minimize testicular tissue damage caused by testicular biopsy.

According to the relevant literature we reviewed, this study focused on NOA patients with AZFc deletion, and it had the largest number of cases in this patient category in a single-center study. Our findings indicate that micro-TESE treatment is a high-SRR strategy for NOA patients with AZFc deletion, which is in line with the findings of most studies [19, 20]. Furthermore, we discovered that age, testicular volume, serum FSH levels, LH levels, and T levels in NOA patients with AZFc deletion may have little bearing on the micro-TESE outcome. Eken et al. found results that were similar to ours [21]. The meta-analysis found that neither serum FSH levels nor testicular volume predicted the micro-TESE SRR, with testicular pathology biopsy being the greatest predictor, confirming our findings [22]. However, NOA patients with elevated FSH levels (including those with Klinefelter syndrome) were shown to have a greater micro-TESE SRR, indicating active spermatogenesis in the testis [23]. The mean testicular volume of men without sperm retrieval after undergoing micro-TESE was 5.7 ml, which was lower than that of men with sperm retrieval, suggesting that a smaller testicular volume is linked to more severe spermatogenic damage [24]. This discrepancy in the results could be due to various study populations or differing micro-TESE sperm retrieval success rates at different medical sites. Even in small testes, areas of normal spermatogenesis may exist, and more research is needed to confirm this in the evaluation of predictors.

Diagnostic testicular biopsy to determine internal testicular spermatogenesis prior to micro-TESE is a specialty of our center. TESA is performed to obtain sufficient tissue for both microscopic and histological examination. The presence of tubules with spermatozoa in a diagnostic testicular biopsy conducted before micro-TESE was found to be the strongest predictor of surgical sperm retrieval in patients with NOA [25]. This supports our findings that a preoperative diagnostic testicular biopsy detecting sperm improves the micro-TESE SRR. However, the most meaningful findings also revealed that patients who underwent micro-TESE without preoperative diagnostic testicular biopsy had a good SRR (63.8%), with no significant difference from the diagnostic testicular biopsy-negative group (65.1%), implying that preoperative diagnostic testicular biopsy may not be necessary. In our study, some patients (*n* = 22) with a positive TESA had a negative final micro-TESE (*n* = 2), which accounted for 9.09%. This could be because the testicular tissue along the needle path would have been severely damaged during the puncture, which could explain why some patients with positive TESA had negative final micro-TESE [26]. Despite the benefits of TESA, which is minimally invasive and rapid, studies have shown that postoperative problems such as hemorrhage, hematoma development, and infection can impair the micro-TESE outcome and cause unnecessary damage to patients [27].

## Conclusion

We performed a retrospective study to analyze clinical data from NOA patients with AZFc deletion who underwent micro-TESE. Our findings imply that age, testicular volume, FSH levels, LH levels, and T levels in NOA patients with AZFc deletion who underwent micro-TESE did not affect the outcome. Patients with no sperm found in preoperative diagnostic testicular biopsy still have a high chance of sperm retrieval by micro-TESE. Negative diagnostic testicular biopsy outcomes have a poor effect in predicting micro-TESE outcomes in NOA patients with AZFc deletion. Clinicians should carefully consider this group of patients for preoperative diagnostic testicular biopsy to avoid unnecessary trauma.

## Data Availability

The datasets used and/or analyzed during the current study are available from the corresponding author on reasonable request.

## Abbreviations

AZF: Azoospermia factor
EAA: European Academy of Andrology
EMQN: European Molecular Genetics Quality Network
FNA: Fine needle aspiration
FSH: Follicle-stimulating hormone
ICSI: Intracytoplasmic sperm injection
LH: Luteinizing hormone
Micro-TESE: Microdissection testicular sperm extraction
NOA: Non-obstructive azoospermia
SRR: Sperm retrieval rate
T: Testosterone
TESA: Testicular sperm aspiration
